# The Hospital System in South Africa

**Published:** 1913-01-25

**Authors:** 


### THE HOSPITAL SYSTEM IN SOUTH AFRICA.

### Conditions of Work in the Cape Province.

In our issue of January 7, 1911, we discussed the question of hospital administration in South Africa, and commented on the resolutions passed at the South Africa Medical Congress in the previous year. The interesting paper by Sir Kendal Franks, which we published in the same issue, sketched fully the general trend of professional opinion in the Dominion, and showed along what lines local medical men would have supported hospital reform. In the Oversea Dominions, as in all new countries, expert opinion has much greater chance of influencing the decision on questions of national health than in conservative communities where the layman is jealous of the expert, and deems himself fully capable of deciding the most important matters.

The newest Hospital Ordinance is that referred to last week, of the Province of the Cape in the Union of South Africa, which was drawn up by Dr. E. N. Thornton in consultation with the other expert advisers of the Administrator, submitted to the Provincial Council this year, and recently passed, practically without amendment to its main principles.

The first hospital at the Cape was erected on the foreshore in 1656. This was found inadequate, and in 1694 a hospital able to accommodate "500 patients and 750 in an emergency" was built somewhere on the site of the present St. George's Cathedral. A third hospital was built in 1772 close to the Parade Ground, but was afterwards converted into a barracks. In 1816 the Somerset Hospital was founded by Dr. Bailey: this is now a mental hospital, its place having been taken, so far as acute cases are concerned, by the new Somerset Hospital, which, erected in 1862, occupies a fine site near the docks. The King William's Town Hospital was started in 1856, and other provincial towns followed suit; so that to-day there is ample hospital accommodation in the main centres, however under-bedded the isolated district towns may be. "Broadly speaking," remarks Dr. Thornton, "the centres in which there are existing hospitals were wisely chosen, although but little discrimination appears to have been exercised in certain instances as to the extent of accommodation needed." The Government did not actively control and erect hospitals, but encouraged local enterprise by judicious grants in aid of proposed buildings and afterwards in aid of maintenance.

### The Effects of State Inspection.

The hospitals are under the control of local boards of management. Their constitutions differ widely. In some there is a special clause providing that Government approval must be obtained to hospital regulations before these regulations become operative. Others enable the hospital property to be alienated without specific Government sanction; but the exact legal position in this respect, as Dr. Thornton observes, has not been tested. "The Government is represented on the boards by one or more *ex officio* members; but otherwise, except indirectly through the grants in aid, exercises no jurisdiction over the conduct of the affairs of the boards." It claims, however, by virtue of its original contributions to the costs of the hospital buildings, the right to inspect each charity at any time it thinks fit, and also the privilege of securing free treatment for ordinary pauper cases. This inspection system, which seems to have been carried out with great tact and judgment, has been of great practical benefit to the hospitals, particularly since the Department takes care that the recommendations of its inspectors are promptly brought to the notice of the hospital authorities, and action taken thereon. It is interesting to notice that the expenses of hospital office administration are low. Many of the hospitals have medical advisory committees composed of the medical staff, the chairman of which is *ex officio* a member of the board of management.

Recently there has been a decided improvement in the general standard of nursing in all the hospitals, though even now there is room for reform when we learn that in some institutions poisons are not kept in a separate locked cupboard, and probationers in one hospital at least are allowed to give hypodermic injections to patients. Most of the boards have official visitors appointed from the members of the committee or (what strikes us as an excellent method) from among interested subscribers. These visitors submit written reports, and the system seems to be a useful one. With regard to the admission of patients Dr. Thornton notes the abuse of letters and condemns the practice of excluding phthisical patients. This latter point is an important one with which we cannot adequately deal here, though we consider that Dr. Thornton's arguments deserve careful consideration. At all hospitals paying patients are taken, but the rules with regard to these vary considerably. All hospitals, too, have out-patient departments which have grown most rapidly in recent years out of all proportion to the increase of the population served.

Dr. Thornton's suggestions for reforms and improvements have received the approval of the practitioners at the Cape, and were embodied in a new Hospital Ordinance which has now passed the Provincial Council and become law. The Ordinance, we understand, affects only the Cape Province. The hospitals of the other provinces are regulated according to local laws and do not come under the new scheme. It is to be hoped that in course of time there will be effected a certain uniformity in hospital administration and hospital politics throughout the Union.

Dr. Thornton remarks that few can be wholly satisfied with the existing system. "No fixed principle has been adopted and hospitals have been unequally treated." Local influence with the Government seems to have been the main factor in deciding whether a hospital obtained a large or a small grant, and so unequal is the assistance afforded by Government that in some cases institutions are seriously crippled. The obvious way out of this difficulty is to provide effective machinery for automatically adjusting the revenues of the hospitals to the requirements of their respective districts. The suggestions made in regard to this provision are interesting and may be briefly summarised. There was a strong feeling that the Government subsidies should be regulated by the amount of treatment received by free patients, so much being paid each day for every non-paying patient receiving treatment. The great objection to this suggestion is that it provides no limit to the Government's financial liability. To make ends meet the Government rate would have to be fixed at not less than 4s. per day per patient. Secondly, as Dr. Thornton points out, such a system would tend to make hospital managers lax in inquiring into the circumstances of non-paying patients and open the door even wider to abuse. An alternative suggestion was that the Government should pay a *pro rata* rate for every patient who could not pay more than 3s. 6d. per day. There are equal objections to this scheme. A third suggestion is that the grants should be reallocated annually according to the number of free patients dealt with during the year. Dr. Thornton shows statistically how such a system would adversely affect some hospitals and unduly benefit others. Equally unsatisfactory are the suggestions that the grants should be based on the total accommodation provided at the hospitals (since several hospitals are overbedded, and the number of beds rarely adequately represents the actual district requirements), and that the grant should be calculated on the basis of the number of free beds reserved (since in the smaller hospitals beds are interchangeable and not specially reserved). After considering all the suggestions, it was decided, and is now fixed by the Ordinance, that the Government grants shall be awarded on the following scale: Not exceeding twenty-five shillings for every pound subscribed; £ for £ for fees received from paying patients, and £ for £ for sums specially bequeathed for approved purposes, provided that no single grant in this respect exceeds £500; and, finally, one half of the deficit—if any—the remaining half being borne by the local authorities. All hospitals in one district are to be brought under one board of management. This scheme is much more liberal than that provided for under the New Zealand and Australian Ordinances, and certain obvious objections can be made against it. The first and strongest objection is that this liberal provision, especially with regard to wiping out the annual deficit, will tend to make hospital boards lax in soliciting help from the public; in other words, that the scheme will seriously imperil the existence of the voluntary principle. In the Ordinance, however, provision is made for ensuring that the boards shall make reasonable efforts to obtain revenues from voluntary sources and from paying patients. The Government assistance in wiping out the deficit is only to be given if the hospital can show that it has worked and collected a "reasonable sum" in aid. On the other hand, the exceedingly liberal scale of grants in aid apportioned on the amounts raised by subscriptions will tend to encourage boards to get as many subscribers as possible. Considerable objection appears to have been raised with regard to grants in aid apportioned on the amounts received from paying patients, it being alleged that this would tend to make the hospitals compete with private nursing homes. We agree with Dr. Thornton that the danger of unfair competition is small, and that the system will tend to deter patients able to pay from abusing the benefits of the hospital. During the discussions on the Ordinance in the Provincial Council strong objection was raised to the proposal that the local authority should pay part of the deficit contribution. This objection has little weight, since the benefit which the local authority derives from the vast majority of hospitals in the Cape Province is so great that the expenditure should be a good investment.

In each hospital district a hospital board is to be constituted, consisting of not more than twenty-four and not fewer than six members appointed as follows: One-third by the local authorities, one-third by subscribers, one-sixth by the honorary medical staff, and one-third by the Government through the Administrator. Where there is more than one hospital in a district subsidiary committees of management would be established, nominated by the board and by the medical staff of the various hospitals. Power is given to the Government to establish a hospital in a new district if asked to do so by petition signed by at least twenty-five registered voters, and after all concerned have had a chance of noting objections. In the event of a counter-petition being presented, the hospital can only be established by consent of the Provincial Council.

The new boards have their powers and responsibilities pretty carefully defined. They take over all assets and liabilities of the old boards, with special safeguards so far as existing trusts are concerned. They control existing institutions, and may, with the approval of the Government, establish new hospitals or close old ones. They have authority to sell land and buildings and to raise loans and incur temporary overdrafts to meet ordinary recurrent expenditure. Their powers, in fact, are similar in all respects to those held by any board of management of a voluntary hospital in this country, except in the matter of finance. Every board must furnish the Government with an annual estimate for scrutiny; Government approval must be obtained before a loan is raised and to all leases or sales of immovable property. The books of the hospital are to be audited half-yearly by local auditors and once every year by the Government auditor. The Government has the right at any time to inspect any hospital or building belonging to the board and to veto the erection of new buildings. It has the right to make suggestions with regard to the administration and the appointment of responsible hospital officers, but it has no control whatever in these matters, and its function is purely an advisory one. On the other hand, it may withhold its annual grant to an institution which neglects to act upon the recommendations of its inspectors or does not follow the rules of the Ordinance.

